# Impacts of Winter and Spring Water Masses on Demersal Fish Community Structure Around Hainan Island

**DOI:** 10.3390/ani16050809

**Published:** 2026-03-05

**Authors:** Boran Qin, Jiani Dong, Jiajie Chen, Yuange Chen, Wei Tian, Xiaodong Wang, Junsheng Zhong

**Affiliations:** 1 Shanghai University Key Laboratory of Marine Animal Taxonomy and Evolution, Shanghai Ocean University, Shanghai 201306, China; qinbr55@163.com (B.Q.);; 2 East China Sea Fisheries Research Institute, Fisheries Science of Chinese Academy, Shanghai 200090, China

**Keywords:** community structure, distribution, fishes, Hainan Island, water mass

## Abstract

To elucidate water mass impacts on fish diversity in Hainan Island, bottom trawl surveys were conducted at 50 stations around the Island in December 2023 (winter) and April 2024 (spring). K-means clustering identified three water masses: Coastal Water (CW), Mixed Water (MW), and Offshore Water (OW). A total of 396 species were collected. Winter communities demonstrated clear habitat specificity, with distinct dominant species in each water mass (OW, MW, and CW). Conversely, the spring intrusion of warm, saline water facilitated the dominance of migratory species in the MW. Diversity centers shifted significantly northward and shoreward, transitioning from the MW region in winter to the CW region in spring. These findings highlight the critical influence of seasonal hydrodynamics on fish community structure, providing essential baselines for regional fisheries management and conservation.

## Simple Summary

Fish populations are highly sensitive to changes in the ocean environment. This study investigated how different “water masses”—large bodies of water with distinct temperature and salinity levels—affect fish diversity around Hainan Island. By conducting surveys in winter and spring, we analyzed which fish species were present and how their distribution shifted with seasonal water changes. We found that the movement of water masses significantly dictates where fish live, and which species are most abundant. For instance, fish communities in winter were very distinct across different areas, whereas in spring, the intrusion of warmer, saltier water brought more migratory species into mixed water zones. Additionally, the areas with the highest fish diversity moved northward from winter to spring. Understanding these seasonal patterns is vital for protecting marine biodiversity. This knowledge helps fishery managers and conservationists make better decisions to ensure sustainable fishing and healthy ocean ecosystems in the face of environmental changes.

## 1. Introduction

Hydrographic characteristics and water mass dynamics are fundamental drivers in shaping the spatial distribution and community structure of marine organisms [1,2]. To effectively analyze these hydrological environments, the K-means clustering algorithm has been widely adopted as a robust classification tool. Previous applications in adjacent regions have demonstrated the efficacy of this method. For instance, studies in Daya Bay [3], the Pearl River Estuary [4], and the broader South China Sea [5] showed that K-means clustering can reveal the coupling relationships between water mass properties and biological distributions. However, the specific environmental mechanisms governing demersal fish patterns around Hainan Island remain to be fully elucidated. Therefore, understanding these dynamics is indispensable for formulating effective resource restoration strategies and ensuring the long-term sustainability of marine ecosystems.

Hainan Island represents an ideal model system to investigate these mechanisms. It is characterized by an intricate, sinuous coastline extending 1855.27 km, separated from the Leizhou Peninsula to the north by the Qiongzhou Strait and bordered by the Beibu Gulf to the west [6]. Its seafloor topography and complex hydrological conditions have fostered diverse fish communities, establishing these coastal waters as one of the most critical fishing grounds in the South China Sea [7]. Hainan Island is also widely acknowledged as a biodiversity hotspot, characterized by its exceptionally rich plant and animal resources [6,7,8]. Consequently, the fishery industry constitutes a cornerstone of the regional economy. However, the depletion of fishery resources has emerged as a critical crisis. Evidence shows that among China’s 52 traditional offshore fishing grounds, approximately 77% have experienced the disappearance of seasonal “fish floods” (peak fishing periods), and over 80% no longer support the formation of concentrated shoals [9]. Furthermore, human activities [10,11], climate change [12,13] have posed significant threats to local fish migration patterns and habitats.

**Figure 1:**
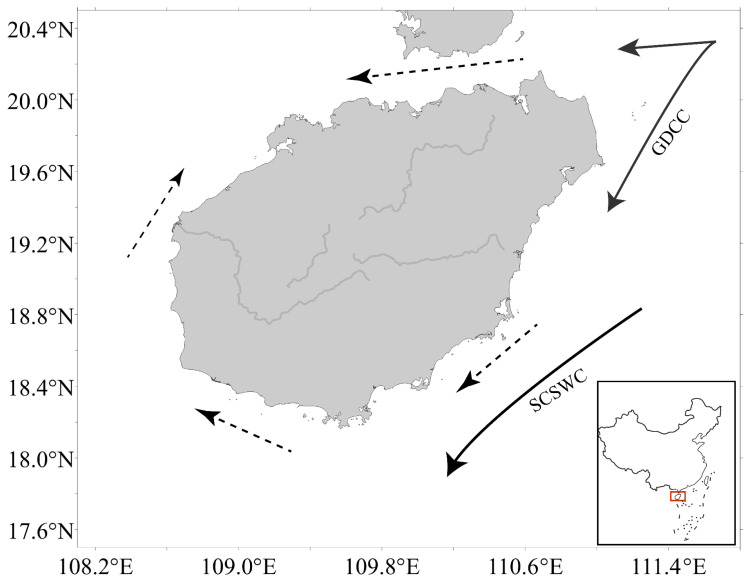
Schematic representation of major surface currents and peripheral circulation around Hainan Island. Solid arrows indicate the major currents, including the Guangdong Coastal Current (GDCC) and the South China Sea West Current (SCSWC). Dashed arrows represent the peripheral currents.

A schematic map of ocean currents around Hainan Island during winter and spring was drawn based on Su [14] (Figure 1). Specifically, the Guangdong Coastal Current (GDCC) (solid arrows) carried cold coastal water southward, South China Sea West Current (SCSWC) mainly affects the western and southern regions, while the peripheral circulation (dashed arrows) mediated the expansion of mixed water in the Qiongzhou Strait and the western Beibu Gulf.

Based on bottom trawl surveys conducted in Winter 2023 (December) and Spring 2024 (April) in the coastal waters of Hainan Island, this study examined how water masses influence fish community composition, dominant species, and biodiversity. Two hypotheses were tested: (1) Different water masses harbor distinct fish communities due to variations in temperature and salinity; (2) Fish community structure shifts in response to seasonal water mass dynamics. The goal is to elucidate the environmental mechanisms driving the spatio-temporal distribution of fish communities around Hainan Island, thereby providing scientific support for the conservation and sustainable management of local fish resources.

## 2. Materials and Methods

### 2.1. Study Area

**Figure 2:**
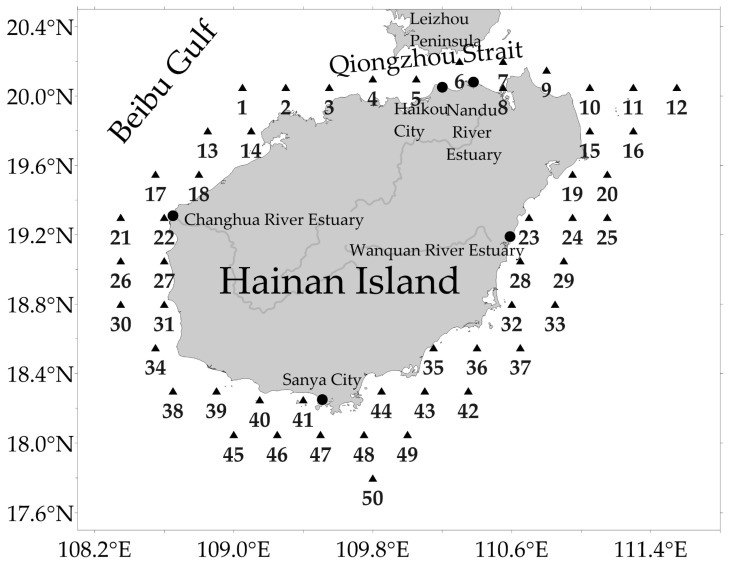
Sampling stations in the surrounding waters of Hainan Island. The triangle represents the Sampling stations. Solid circles represent landmarks.

The study area was located between 108.35 and 111.55° E and 17.80–20.20° N. 50 stations were established along the coastal waters of Hainan Island at intervals of 0.25° longitude and 0.25° latitude (Figure 2) after conducting a preliminary analysis of the marine geography and location of Hainan Island’s nearshore waters. They effectively cover areas ranging from river-influenced estuarine zones and coastal current-influenced strait regions to the open ocean. Sampling stations provide sufficient spatial coverage to capture the heterogeneity of fish community structures across different water masses.

### 2.2. Water Mass Division

Following the methodology of Tian et al. [4], the sampling stations were clustered into three categories using the K-means algorithm based on four parameters: sea surface temperature (SST), bottom sea temperature (BST), sea surface salinity (SSS), and bottom sea salinity (BSS). The goal of K-Means is to minimize the sum of squared distances between each data point and the centroid (mean) of its assigned cluster. This is captured by the formula:

(1) $$J=\overset{C}{\underset{k=1}{\sum }}\sum_{r=1}^{m_{k}}{\left(x_{r}-{\bar{\chi }}_{k}\right)}^{2}$$

In this formula, *J* represents the objective function to be minimized to ensure cluster cohesion. *C* denotes the total number of clusters, *m_k_* represents the number of samples contained in the *k*-th cluster, *r* is the re-indexed sequence number of a sample within the *k*-th cluster, *x_r_* is the observation vector of the *r*-th station, ${\bar{x}}_{k}$ is the mean of the samples in the *k*-th cluster.

### 2.3. Sample Collection and Identification

Field surveys were conducted across two seasons: winter (28 November 2023–12 December 2023) and spring (3–13 April 2024). Bottom trawlings were performed using a single-vessel trawl (net height: 3.0 m; width: 21.0 m; minimum cod-end mesh size: 2.5 cm). Each haul lasted approximately 0.5 h, with an average trawling speed of 3.3 knots. The fishery resource survey was conducted using the Research Vessel “Qionglinyu 12368#”. Captured fish were identified to the species level based on Wu and Zhong [15], and both abundance and biomass densities were calculated for each haul. Ecological types for each species were determined with reference to FishBase [16] and Chen [17]. Warm-water (WW) fishes include those distributed in tropical and subtropical zones, while warm-temperate (WT) fishes refer to those distributed in temperate zones. The systematic arrangement of taxa followed the classification systems of Betancur [18] and Nelson [19]. Physicochemical parameters, including temperature, salinity, pH, and dissolved oxygen (DO), were measured synchronously in the surface and bottom water at each site using YSI ProDSS Multiparameter Digital Water Quality Meter (YSI Inc., Yellow Springs, OH, USA). Depth data were concurrently collected using an onboard echo sounder.

### 2.4. Estimates of Fish Resource Density

The resource density at each sampling station was estimated using the swept area method [20]. The calculation formula is as follows:

(2) $$p_{i}\;=\;\frac{C_{i}}{\left(a_{i}q\right)}$$

*p_i_* denotes the fish biomass or abundance, measured in kg/km^2^ or ind./km^2^; *C_i_* represents the catch per hour at the *i*-th station (kg/h or ind./h); *a_i_* is the swept area per hour of the fishing gear at the *i*-th station (km^2^/h); *q* is the catchability coefficient. Following standard procedures, the catchability coefficient (*q*) for demersal fish was set to 0.5 [21].

### 2.5. The Index of Relative Importance (IRI)

The ecological dominance of fish communities during different seasons was evaluated using the index of relative importance (*IRI*) [22]:

(3) IRI = (N + W) × F × 10,000

The *IRI* is computed by considering three factors: *N* represents the percentage of individuals of a particular fish species relative to the total catch number; *W* represents the percentage of the weight of that fish species relative to the total catch weight; and *F* represents the percentage of occurrences of that fish species relative to the total number of sampling stations. Species with an *IRI* ≥ 1000 are categorized as dominant species, species with *IRI* between 100 and 1000 are classified as important species, species with *IRI* between 10 and 100 are considered common species, and species with *IRI* < 10 are regarded as rare species [21].

### 2.6. Linear Regression Analysis

Simple linear regression analysis was performed to determine the relationship between the abundance/biomass density of species and the total density of the fish community [23]. The standardized regression coefficient (*β*) was used to evaluate the contribution rate of each species to the variation in total stock density. Data were analyzed using IBM SPSS Statistics 26.

### 2.7. Alpha Diversity Used Indices

The Shannon-Wiener diversity index (*H*′), Margalef species richness index (*D*), and Pielou evenness index (*J*′) were employed to evaluate the abundance-based diversity of the fish community [21].

Shannon-Wiener diversity index (*H*′):

(4) $$H^{\prime }=-\sum_{i=1}^{s}\left(P_{i}lnP_{i}\right)$$

Margalef species richness index (*D*):

(5) $$D=\frac{(S-1)}{\ln N}$$

Pielou evenness index (*J*′):

(6) $$J^{\prime }=\frac{H^{\prime }}{\ln S}$$

In the formula, *S* represents the total number of species of captured fish in the study area, *P_i_* denotes the weight of the *i*-th fish species, *N* represents the total number of individuals of captured fish.

### 2.8. ANOSIM and SIMPER Analysis

We utilized cluster analysis to investigate the community structure based on the Bray–Curtis dissimilarity. One-way analysis of similarities (ANOSIM) [24], was employed to evaluate the significance of variations in community structure among different water masses, based on abundance and biomass data. Prior to analysis, both abundance and biomass data underwent square-root transformation to mitigate the influence of highly dominant species. Additionally, similarity percentage (SIMPER) analysis was conducted to determine the contribution of specific species to intra-group similarity and inter-group dissimilarity within the community structure [25]. All computational analyses were performed using Primer 6.0.

## 3. Results

### 3.1. Classification and Properties of Water Masses

**Figure 3:**
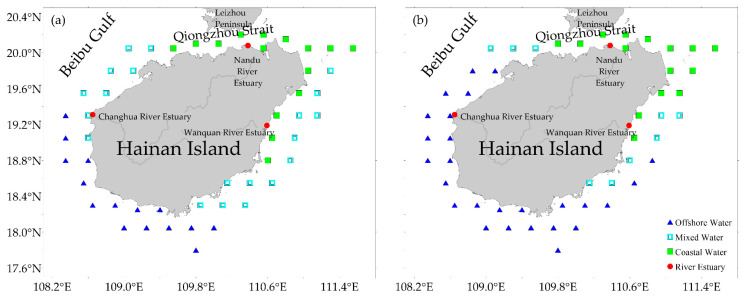
Distribution of water masses based on integrated water column characteristics. (**a**) is for winter; (**b**) is for spring.

The waters across both seasons were classified into three water masses: Coastal Water (CW), Mixed Water (MW), and Offshore Water (OW) (Table 1, Figure 3). The identified water masses exhibited distinct hydrographic signatures. The CW, characterized by relatively low temperature and salinity, was primarily distributed in the northeastern waters of Hainan Island, spanning from the Wanquan River estuary to the Qiongzhou Strait. Conversely, the OW featured high-temperature and high-salinity profiles, predominantly localized in the southern and western sectors from the vicinity of Sanya to the Changhua River estuary. The MW, representing the transitional zones between high-salinity offshore water and coastal water, showed intermediate values with pronounced salinity fluctuations, mainly occupying the southeastern and northwestern regions (Figure 3b).

Overall, both temperature and salinity were higher in spring than in winter. OW exhibited the highest thermal stability, characterized by the smallest standard error of the mean in spring. In the CW, the average SSS rose from 33.07 in winter to 33.93 in spring. The temperature and salinity of OW remained uniform throughout the water column in both seasons, maintaining its characteristic high-temperature and high-salinity profile. The spatial distribution of water masses also varied between the two seasons.

Winter featured 20 MW stations and 15 OW stations, whereas spring had 9 MW stations and 26 OW stations. This indicates a strengthened influence of OW in spring, which occupied more transitional stations.

### 3.2. Specific Composition

**Figure 4:**
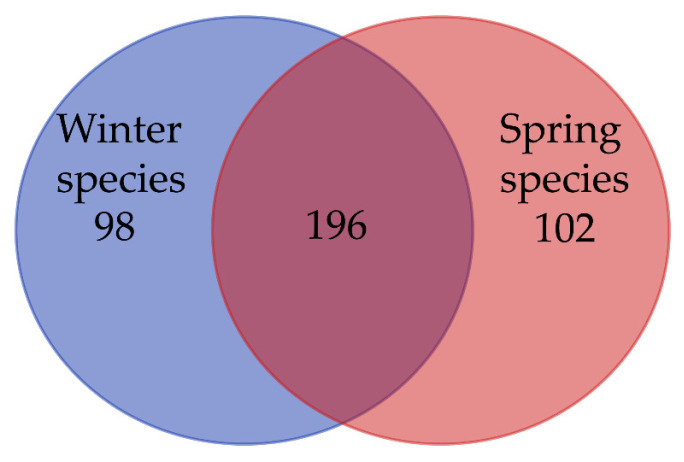
Venn diagram of seasonal species distribution. Blue represents winter species; Red represents spring species; Overlap represents shared species.

**Figure 5:**
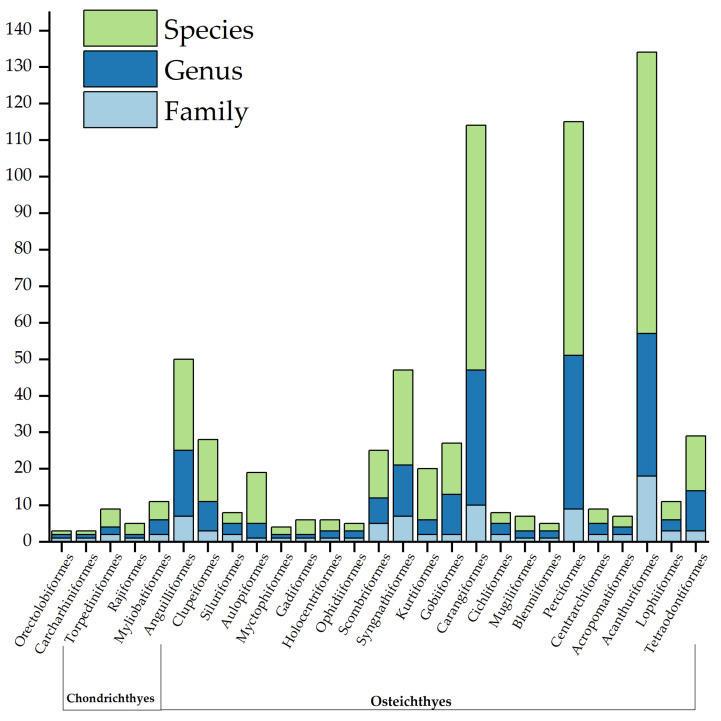
Taxonomic composition of the demersal fish community at different hierarchical levels (Order, Family, Genus, and Species) in the coastal waters of Hainan Island. The bars represent the number of taxa within each fish Order identified during the winter and spring. The horizontal axis categorizes fish into two major classes: Chondrichthyes and Osteichthyes.

The total catch comprised 56,960 individuals, distributed across 396 species, 228 genera, 91 families, and 27 orders (Table A1). A substantial overlap of 196 shared species was observed between the two seasons, whereas the number of species occurring exclusively in winter and spring was 98 and 102, respectively (Figure 4). At the Order level, Acanthuriformes was the most speciose order with 77 species, accounting for 19.44% of the total number of species, followed by Carangiformes (67 species), Perciformes (64 species), and Syngnathiformes (26 species) (Figure 5). Seasonally, the winter survey yielded 26,253 individuals across a total number of 294 species, while spring saw an increase in both numbers (30,707 individuals) and the number of species (298). 

**Figure 6:**
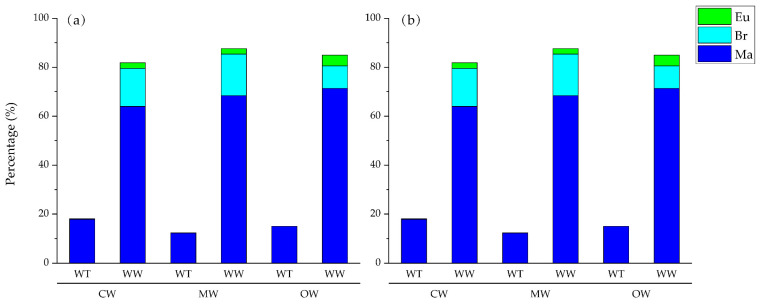
Composition of ecological groups of fishes of warm-temperate (WT), warm-water (WW), marines (Ma), brackish (Br), euryhaline (Eu) in Water Masses of Coastal Water (CW), Mixed Water (MW), and Offshore Water (OW). (**a**) is for winter; (**b**) is for spring.

In winter, WT species totaled 31, comprising 29 marine (Ma) species, 1 brackish (Br) species, and 1 euryhaline (Eu) species. Simultaneously, WW species reached 263, including 181 Ma species, 61 Br species, and 21 Eu species. During the spring survey, the WT species numbered 30, comprising 27 Ma species, 2 Br species, and 1 Eu species. Simultaneously, WW species reached a total of 268, which included 182 Ma species, 61 Br species, and 25 Eu species. Across both seasons, WT species and Ma species remained the dominant components within all water masses, consistently accounting for over 50.00% of the total fish community composition (Figure 6). The CW region experienced the most dramatic seasonal shifts; in winter, the proportion of Eu species reached 25.48%. As salinity increased in spring, the Eu proportion plummeted to 2.34%. Conversely, the ratio of WT-Ma species surged from 4.60% in winter to 17.94% in spring. In winter, the MW was co-dominated by WW-Ma (56.58%) and WW-Br (24.20%) species. In spring, the dominance of WW-Ma species in the MW further strengthened to 68.38%, while Eu species nearly disappeared; the species composition in this transitional zone showed a clear successional trend toward OW characteristics. The OW remained the most environmentally stable with the strongest marine affinity; WW-Ma groups accounted for over 70% in both seasons, and proportions of Eu and Br groups were minimal in the OW, with even lower values recorded in spring than in winter.

### 3.3. Seasonal Variations in Fish Resource Density

**Figure 7:**
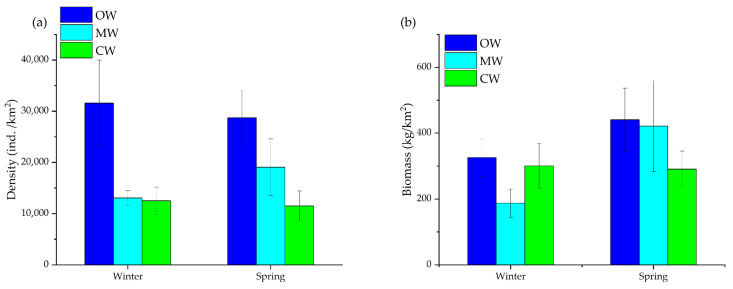
(**a**) Density and (**b**) Biomass in water masses of Coastal Water (CW), Mixed Water (MW), and Offshore Water (OW).

The average fish biomass density in both seasons was 335.16 kg/km^2^. Biomass density consistently peaking in the OW and remaining lowest in the MW during winter. A Rise in biomass density was observed in spring, particularly in the OW region (Figure 7). The maximum density at a single station was 854.63 kg/km^2^, located in the northern CW region, with *Johnius taiwanensis* (418.94 kg/km^2^) as the most abundant species. In spring, a high-density area emerged in the transition zone between southeastern OW and MW, where the maximum station density was 2437.43 kg/km^2^, mainly by the presence of *Thamnaconus hypargyreus* (1371.37 kg/km^2^).

The average abundance density of fish across both seasons was 20,235.75 ind./km^2^. Fish abundance showed clear seasonal changes. Across both seasons, OW consistently supported the highest densities, while CW maintained the lowest. Although average abundance increased in spring, the spatial contrast between the high-density OW and low-density CW remained, with OW still hosting the most fish and CW the least. (Figure 7). During winter, a high-density area was identified in the southern OW region, where the maximum station was 103,918.22 ind./km^2^, by the presence of *Alepes kleinii* (3869.08 ind./km^2^). In spring, high-density patches emerged in the transition zone between southeastern OW and MW, with the maximum density reaching 128,134.11 ind./km^2^, by the presence of *Saurida undosquamis* (12,125.35 ind./km^2^).

### 3.4. Seasonal Variation of Dominant and Key Contributing Species

Although *Acropoma japonicum* exhibited the highest *IRI* values (919.13), no single species met the strict threshold for dominance (*IRI* ≥ 1000). However, 14 important species were recorded, with 1 Eu species, 5 Br species, and the remainder as Ma species. In spring, *S. undosquamis* emerged as the only dominant species. Additionally, 12 important species were identified, with only 1 Br species and the rest being Ma species. Common dominant or important species across both seasons included *S. undosquamis*, *Champsodon snyderi*, *Brachypleura novaezeelandiae*, *A. japonicum*, *Decapterus kurroides*, *Upeneus japonicus*, *Ostorhinchus kiensis*, *Terapon theraps*, and *Pennahia macrocephalus* (Table 2).

### 3.5. Key Contributing Species in Water Masses

**Figure 8:**
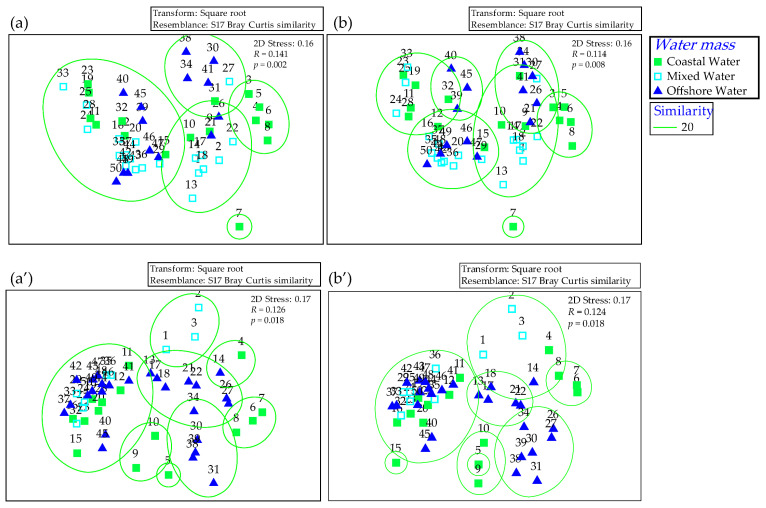
NMDS ordination of sampling stations from three water masses of Coastal Water (CW), Mixed Water (MW), and Offshore Water (OW) based on Bray–Curtis similarity of square-root transformed biological data. (**a**,**b**) Winter; (**a’**,**b’**) Spring. (**a**,**a’**) Individual density; (**b**,**b’**) Biomass.

**Figure 9:**
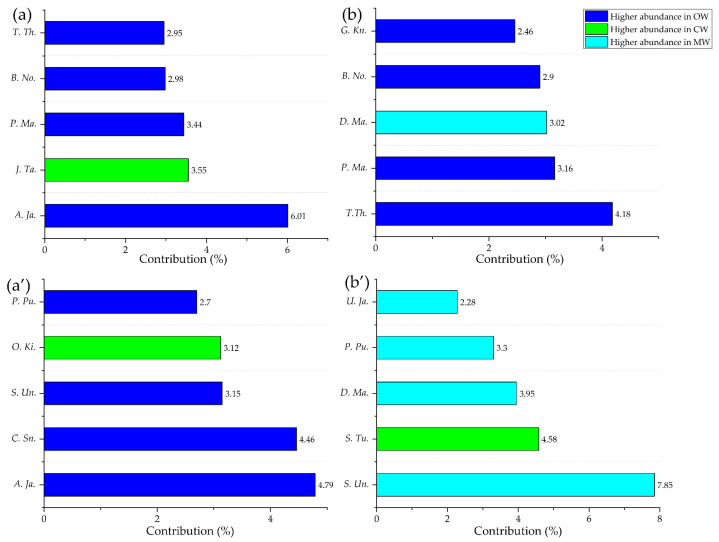
Bar plots of SIMPER analysis showing the top five species contributing most to community dissimilarity, for the pairwise water masses of Coastal Water (CW), Mixed Water (MW), and Offshore Water (OW) comparison with the most pronounced dissimilarities among all seasons. (**a**) winter density (OW vs. CW); (**b**) winter biomass (OW vs. MW); (**a’**) spring density (OW vs. CW); (**b’**) spring biomass (CW vs. MW). *T. Th.*: *Terapon theraps*; *B. No.*: *Brachypleura novaezeelandiae*; *P. Ma.*: *Pennahia macrocephalus*; *J. Ta.*: *Johnius taiwanensis*; *A. Ja.*: *Acropoma japonicum*; *G. Kn.*: *Grammoplites knappi*; *D.Ma.*: *Decapterus maruadsi*; *P. Pu.*: *Paramonacanthus pusillus*; *O. Ki.*: *Ostorhinchus kiensis*; *S. Un.*: *Saurida undosquamis*; *C. Sn.*: *Champsodon snyderi*; *U. Ja.*: *Upeneus japonicus*; *S. Tu.*: *Saurida tumbil*.

Water masses had a significant influence on the spatial distribution patterns of fish communities in both seasons (ANOSIM, *p* < 0.05) (Figure 8). SIMPER analysis revealed distinct variations in community composition across different water masses (Figure 9).

In winter, the primary contributors to abundance in MW (contribution > 10%) were *B. novaezeelandiae* (11.07%) and *C. snyderi* (10.01%), with *B. novaezeelandiae* (12.24%) also being the main biomass contributor. In OW, *A. japonicum* (10.44%) was the main abundance contributor, while *T. theraps* (12.79%) dominated the biomass contribution. In CW, *J. taiwanensis* was the main contributor to both abundance (14.80%) and biomass (14.40%). In spring, the main abundance contributors in MW were *S. undosquamis* (11.82%) and *C. snyderi* (10.60%). Biomass was primarily contributed by *S. undosquamis* (19.84%), *D. maruadsi* (14.98%), and *S. tumbil* (13.70%). In OW, *A. japonicum* (10.40%) remained the dominant abundance contributor, whereas *S. undosquamis* (14.40%) and *S. tumbil* (11.24%) were the main biomass contributors. In CW, *J. taiwanensis* (14.40%) led the abundance contribution, while *S. tumbil* (17.91%) became the primary contributor to biomass.

### 3.6. Drivers of Community Succession

To further identify the primary drivers of community succession, this study compared the contributions of the top five dominant species and the aforementioned key water mass contributors to community biomass (*W*) and abundance (*N*). Multiple linear regression analysis revealed a significant seasonal turnover in the species driving variations in fish community composition.

In winter, the total *W* was jointly driven by multiple species. Specifically, *J. taiwanensis*, *T. theraps*, and *D. maruadsi* exhibited highly significant positive contributions (*p* < 0.01), serving as the core factors determining winter biomass distribution patterns. In contrast, variations in winter total *N* were significantly correlated only with *A. japonicum* (*p* < 0.05). In spring, the driving force of the community shifted markedly toward marine species; *S*. *undosquamis* became the primary factor influencing abundance (*p* < 0.01), with a standardized coefficient (*β*) of 0.39. These results indicate an overlap between the top five dominant species and the major water mass contributors: *J. taiwanensis* and *S. undosquamis*, which collectively drive the spatial heterogeneity of the community. However, certain dominant species, such as *A. japonicum* in winter, primarily exerted influence at the numerical level, with relatively limited impact on the biomass distribution patterns (Table 3).

### 3.7. Seasonal Variations in Diversity

**Figure 10:**
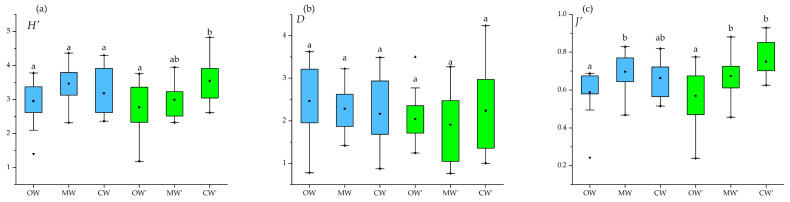
Diversity indices across seasons (OW, Mw and CW for Winter; OW’, MW’ and CW’ for Spring). Maximum, minimum, and average values are indicated by a black triangle, black inverted triangle, and black square, respectively. Different lowercase letters in the figures represent the significant differences at *p* < 0.05. (**a**) is for Shannon-Wiener diversity index (H), (**b**) is for Margalef’s richness index(D), (**c**) is for Pielou’s evenness index (*J*′). Blue bar represents winter, Green bar represent spring.

Fish community diversity exhibited distinct seasonal variations. In winter, high-value zones for the Margalef richness index (*D*) were primarily concentrated in the high-salinity OW region. However, high-value zones for the Shannon–Wiener diversity index (*H*′) and Pielou’s evenness index (*J*′) were mainly distributed in the confluence area between CW and MW. The Pielou’s evenness index (*J*′) in the MW was significantly higher than in the OW (*p* < 0.05). This pattern may be attributed to the dominance of a single abundance contributor, *A. japonicum*, in the OW region, which resulted in relatively lower evenness. In spring, the centers of community diversity displayed a distinct northward and shoreward shift. High-value zones for the Margalef’s richness index (*D*) transitioned to the CW and MW regions in eastern Hainan Island, with the highest number of species in the CW. Simultaneously, the Shannon–Wiener diversity index (*H*′) and Pielou’s evenness index (*J*′) peaked in the CW region near northeastern Hainan Island and the Qiongzhou Strait. Both indices were significantly higher than those in the OW (*p* < 0.05) (Figure 10).

## 4. Discussion

### 4.1. Fish Composition

The fish community around Hainan Island is characterized by high species richness and a clear dominance of warm-water marine (WW-Ma) groups. The identification of 396 species across both seasons aligns with previous records from the South China Sea shelf [21]. However, the community structure undergoes a significant reorganization driven by seasonal water mass shifts. The key species driving total biomass and abundance distributions are highly congruent with the core taxa defining specific water masses.

In winter, the spatial heterogeneity of biomass is jointly regulated by a multispecies complex. Notably, *J. taiwanensis* mainly occurs in shallow tropical and subtropical coastal waters [26]. It serves as a high-fidelity indicator for Coastal Water (CW), with its density peaks strictly confined to the low-temperature, low-salinity boundaries of the Qiongzhou Strait. Conversely, *A. japonicum* acts as a representative taxon for OW region. Primarily concentrated in the high-salinity waters off Sanya City. This is consistent with its habit of preferring areas of relatively deep water with relatively high salinity [27]. In contrast, the spring transition marks a shift toward a community driven by migratory marine species. The intrusion of warm OW region into transitional zones facilitates the dominance of *S. undosquamis*, which becomes the primary factor influencing regional abundance. The proliferation of *S. undosquamis* is linked to spawning migrations [28]. Furthermore, the seasonal strengthening of the SCSWC likely facilitates larval dispersal and connectivity [29]. The synchrony between species turnover and water mass dynamics suggests that these key taxa act as reliable bioindicators of environmental fluctuations. This environmental dependency highlights the role of water masses regulate fish distribution through the selection of species with specific thermohaline adaptations. From a biodiversity perspective, this seasonal habitat partitioning enhances ecosystem resilience by promoting niche differentiation. This temporal segregation allows for greater species coexistence and functional redundancy, buffering the Hainan Island fishery ecosystem against local disturbances.

### 4.2. The Influence of Water Masses on the Distribution of Fish Species

The waters surrounding Hainan Island are primarily influenced by three types of water masses. Shi et al. [30] identified a year-round westward flow in the Qiongzhou Strait, with a near-bottom average flow rate of 10–15 cm/s during winter and spring. The Guangdong Coastal Current carries a significant volume of supplementary water westward through the strait, while freshwater from the Wanquan and Nandu Rivers discharges into the northeastern region. These combined processes give rise to distinctive CW characteristics in and around the Qiongzhou Strait. The SCSWBC generates coastal currents adjacent to the eastern coast of Hainan Island, flowing southward in winter and northward in summer, with the winter flow being stronger than that in summer [31]. During winter and spring, a clockwise circulation develops around Hainan Island [32], resulting in a stronger influence of high-salinity South China Sea water in the southwestern region. The MW is positioned between these two water masses. During the spring monsoon transition, the influence of high-salinity water strengthens, leading to a contraction of the MW. In winter, the contributing species of each water mass exhibit high specificity: the euryhaline *J. taiwanensis* is strictly confined to the Qiongzhou Strait dominated by CW, while the marine warm-water species *A. japonicum* is concentrated in the waters around Sanya City covered by high-salinity water. A weakening of the SCSWBC is observed in spring [33,34], allowing southern OW region to migrate northward and mix with the coastal water in the eastern region. With the arrival of warm OW region, migratory species such as *S. undosquamis* and *S. tumbil* complete their overwintering and aggregate in large numbers near the Wanquan River estuary for spawning [29]. These species replace the *B. novaezeelandiae* and *C. snyderi* as the main abundance contributors to the MW (Table 3). In spring, the strengthening of high-salinity water reduces the extent of MW along both the eastern and western coasts. Meanwhile, marine species like A. japonicum migrate northwestward with the warm water mass. High abundance and biomass density zones, primarily contributed by marine species such as *S. undosquamis*, *D. maruadsi*, and *S. tumbil*, emerge in the southeastern high-salinity and MW regions, forming a distinct spatial asymmetry. The community exhibits a clear trend of warmer and higher salinity (Figure 6). These findings highlight the need for dynamic fisheries management. Spawning sites identified can be used for seasonal closures to protect critical life stages.

### 4.3. The Impact of Water Masses on Diversity

The fish community diversity in the waters around Hainan Island showed pronounced seasonal and spatial variations. In winter, high values of the species richness index (*D*) were concentrated in the high-salinity OW south of the island, indicating that this area serves as an important overwintering ground with considerable species reserves. In contrast, the hotspots of the *H*′ and *J*′ were located farther east, mainly within the convergence zone of CW and MW. Thermohaline fronts in the South China Sea may enhance nutrient transport from shallow shelf waters to offshore areas [35], potentially supporting higher evenness in these transitional zones. Statistically, *J*′ in MW was significantly higher than in OW (*p* < 0.05), while OW exhibited the lowest *H*′ among the three water masses. This pattern reflects the strong dominance of *A. japonicum* in offshore waters during winter [27]. Regression analysis confirmed that *A. japonicum* was the only significant driver of abundance variation in that season; its locally dense aggregations reduced community evenness and thus constrained overall diversity levels.

In spring, the centers of diversity shifted distinctly northward and shoreward. Higher richness areas moved to the CW and MW regions in eastern Hainan Island, with the highest species richness recorded in CW. Simultaneously, significantly elevated *H*′ and *J*′ values were observed in CW near northeastern Hainan Island and the Qiongzhou Strait, both markedly exceeding those in OW during the same period. The seasonal increase in temperature and salinity likely stimulated the inshore migration of Br and Eu species from OW into the lower-salinity MW and CW zones. As conditions change, marine species are colonizing areas that were historically cooler and less saline [36]. Warming water also provides optimal thermal conditions for early life stage development [37]. This influx of species with differentiated ecological niches promoted a more balanced species distribution and ultimately enhanced overall community diversity. The observed diversity shifts reveal coastal ecosystem vulnerability to climate change, underscoring the need to integrate climate adaptation into long-term conservation plans.

## 5. Conclusions

This study demonstrates that the fish community around Hainan Island is predominantly composed of warm-water marine species, with its spatio-temporal structure significantly modulated by seasonal water mass dynamics. In winter, the euryhaline *J. taiwanensis* and the marine *A. japonicum* serve as primary bioindicators for the Coastal Water and Offshore Water, respectively, reflecting strict environmental filtering. The spring monsoonal transition facilitates the intrusion of high-temperature and high-salinity Offshore Water into the Mixed Water region. This hydrographic shift prompts a distinct successional trend where migratory marine species, notably *S. undosquamis*, replace winter species to become the primary drivers of regional abundance and biomass. Driven by these thermohaline fluctuations, the centers of community diversity undergo a significant northward and shoreward migration, transitioning from the Mixed Water toward the Coastal Water regions in eastern Hainan Island. The identified diversity seasonal migration patterns provide a scientific basis for formulating dynamic resource conservation measures and sustainable utilization plans. Further research should focus on specific characteristics of Hainan Island’s water mass, such as nutrient flux and primary productivity.

## Figures and Tables

**Table 1:** Ranges and average values of sea surface temperature (SST), bottom sea temperature (BST), sea surface salinity (SSS), and bottom sea salinity (BSS), in Coastal Water (CW), Mixed Water (MW), and Offshore Water (OW) winter and spring.

Season	Water Mass		SST (°C)	BST (°C)	SSS	BSS
Winter	CW	Range	23.70–25.00	23.60–24.90	31.60–34.40	31.70–34.20
		Average	24.37 ± 0.09	24.39 ± 0.10	33.07 ± 0.20	32.99 ± 0.20
	MW	Range	24.90–26.00	25.00–25.80	30.60–34.00	32.30–34.20
		Average	25.47 ± 0.07	25.40 ± 0.06	33.07 ± 0.19	33.25 ± 0.13
	OW	Range	25.90–27.20	25.90–27.20	32.90–34.20	31.60–34.20
		Average	26.64 ± 0.11	26.61 ± 0.12	33.6 ± 0.10	33.21 ± 0.15
Spring	CW	Range	22.00–35.70	22.20–35.50	31.80–34.90	33.40–35.20
		Average	24.06 ± 0.85	23.95 ± 0.84	33.93 ± 0.22	34.29 ± 0.17
	MW	Range	25.20–27.30	23.60–25.90	33.90–34.80	33.50–34.90
		Average	26.03 ± 0.23	25.04 ± 0.23	34.48 ± 0.13	34.46 ± 0.19
	OW	Range	25.60–28.90	25.90–29.00	33.70–34.80	33.70–34.80
		Average	27.30 ± 0.13	27.23 ± 0.13	34.29 ± 0.05	34.34 ± 0.05

**Table 2:** Community composition, abundance (*N*), biomass (*W*), frequency of occurrence (*F*), and index of relative importance (*IRI*) of dominant and important fish species in different seasons.

Seasons	Species	*W* (%)	*N* (%)	F (%)	*IRI*	Temperature Adaptation	Salinity Adaptation
Winter	*Acropoma japonicum*	1.53	18.45	0.46	919.13	WW	Ma
	*Johnius taiwanensis*	8.11	4.62	0.42	534.59	WW	Eu
	*Decapterus maruadsi*	7.11	3.57	0.5	534.2	WW	Ma
	*Terapon theraps*	6.60	3.07	0.46	444.57	WW	Br
	*Photopectoralis bindus*	0.94	4.61	0.72	399.87	WW	Br
	*Champsodon snyderi*	2.02	3.20	0.76	396.45	WW	Ma
	*Pennahia macrocephalus*	4.18	5.32	0.34	322.98	WW	Ma
	*Brachypleura novaezeelandiae*	2.97	3.94	0.44	304.16	WW	Ma
	*Cynoglossus arel*	3.31	2.77	0.46	279.7	WW	Br
	*Ostorhinchus kiensis*	0.31	4.53	0.54	261.54	WW	Br
	*Upeneus japonicus*	1.75	3.10	0.4	193.99	WW	Ma
	*Saurida undosquamis*	1.49	1.78	0.46	150.41	WW	Ma
	*Deveximentum ruconius*	0.26	2.04	0.56	129.14	WW	Br
	*Trachinocephalus myops*	4.56	1.59	0.18	110.66	WW	Ma
Spring	*Saurida undosquamis*	18.43	8.16	0.56	1488.95	WW	Ma
	*Champsodon Snyderi*	1.52	11.83	0.46	614.01	WW	Ma
	*Saurida tumbil*	5.79	2.11	0.7	552.87	WW	Ma
	*Acropoma japonicum*	1.64	10.87	0.38	475.21	WW	Ma
	*Paramonacanthus pusillus*	2.70	4.41	0.56	397.99	WW	Ma
	*Ostorhinchus kiensis*	0.47	6.28	0.58	391.63	WW	Ma
	*Thamnaconus hypargyreus*	8.21	8.20	0.2	328.11	WW	Ma
	*Upeneus japonicus*	2.75	3.13	0.52	305.76	WW	Ma
	*Decapterus maraudsi*	3.13	2.65	0.42	242.74	WW	Ma
	*Terapon theraps*	4.13	2.08	0.32	198.67	WW	Br
	*Brachypleura novaezeelandiae*	1.18	2.57	0.5	187.5	WW	Ma
	*Pennahia macrocephalus*	3.10	3.57	0.24	159.92	WW	Ma
	*Priacanthus macracanthus*	1.41	1.03	0.42	102.1	WW	Ma

**Table 3:** Summary of multiple linear regression analysis of key fish species contributing to the variation in total biomass density (*W*) and total abundance (*N*) across seasons.

Season	Species	*W*			*N*		
		*β*	*t*	*p*	*β*	*t*	*p*
Winter	*Acropoma japonicum * ^a,b^	0.18	1.14	-	0.38	2.24	*
	*Johnius taiwanensis * ^a,b^	0.52	4.43	**	0.13	0.85	-
	*Decapterus maruadsi * ^a^	0.43	3.34	**	0.24	1.36	-
	*Terapon theraps * ^a^	0.45	3.88	**	0.03	0.19	-
	*Photopectoralis bindus * ^a^	−0.02	−0.22	-	0.15	0.87	-
	*Brachypleura novaezeelandiae * ^b^	−0.03	−0.19	-	0.01	0.04	-
	*Champsodon snyderi * ^b^				−0.11	−0.54	-
Spring	*Saurida undosquamis * ^a,b^	0.43	1.70	-	0.39	1.77	*
	*Champsodon snyderi * ^a,b^	0.15	0.61	-	0.27	1.23	-
	*Saurida tumbil * ^a^	−0.34	−1.36	-	0.02	0.10	-
	*Acropoma japonicum * ^a,b^	−0.19	−1.08	-	−0.17	−1.00	-
	*Paramonacanthus pusillus * ^a^	0.32	1.22	-			
	*Decapterus maruadsi * ^b^	−0.14	−0.87	-			
	*Ostorhinchus kiensis * ^b^				0.01	0.06	-
	*Paramonacanthus pusillus * ^b^				−0.05	−0.16	-
	*Johnius taiwanensis * ^b^				−0.05	−0.39	-

Notes: *β* represents the standardized regression coefficient. *t* represents test statistic. *p* represents significance. ** denotes *p* < 0.01, * denotes *p* < 0.05. Superscript a (^a^) indicates the species is among the top five dominant species according to the *IRI*. Superscript b (^b^) indicates the species is a major contributor to the similarity/dissimilarity of water masses identified by SIMPER analysis (contribution > 10%). Winter *W*: *R*^2^ = 0.486, Adj. *R*^2^ = 0.414, *F* = 6.772, *p* < 0.01, *SE* = 170.77 kg/km^2^. Winter *N*: *R*^2^ = 0.260, Adj. *R*^2^ = 0.137, *F* = 2.110, *p* = 0.063, *SE* = 18,173.18 ind./km^2^. Spring *W*: *R*^2^ = 0.278, Adj. *R*^2^ = 0.178, *F* = 2.763, *p* < 0.05, *SE* = 391.88 ind./km^2^. Spring *N*: *R*^2^ = 0.316, Adj. *R*^2^ = 0.202, *F* = 2.776, *p* < 0.05, *SE* = 20,536.27 ind./km^2^. *R*^2^ = coefficient of determination; Adj. *R*^2^ = adjusted *R*^2^; *SE* = standard error of the estimate.

## Data Availability

The data will be made available by the authors on request.

## Appendix A

**Table A1:** Complete list of collected fish species and their IUCN Red List status.

Class	Order	Family	Genus	Species	IUCN Redlist
Elasmobranchii	Orectolobiformes	Hemiscylliidae	*Chiloscyllium*	*Chiloscyllium plagiosum*	NT
	Carcharhiniformes	Carcharhinidae	*Scoliodon*	*Scoliodon macrorhynchos*	NT
	Torpediniformes	Platyrhinidae	*Platyrhina*	*Platyrhina sinensis*	EN
				*Platyrhina tangi*	VU
		Narcinidae	*Narcine*	*Narcine lingula*	VU
				*Narcine prodorsalis*	EN
				*Narcine timlei*	VU
	Rajiformes	Rajidae	*Okamejei*	*Okamejei acutispina*	VU
				*Okamejei boesemani*	VU
				*Okamejei hollandi*	VU
	Myliobatiformes	Dasyatidae	*Telatrygon*	*Telatrygon zugei*	VU
			*Hemitrygon*	*Hemitrygon akajei*	NT
				*Hemitrygon bennetti*	VU
			*Neotrygon*	*Neotrygon kuhlii*	DD
		Gymnuridae	*Gymnura*	*Gymnura japonica*	VU
Actinopteri	Anguilliformes	Synaphobranchidae	*Dysomma*	*Dysomma anguillare*	LC
		Muraenidae	*Gymnothorax*	*Gymnothorax chilospilus*	LC
				*Gymnothorax minor*	LC
				*Gymnothorax pindae*	LC
				*Gymnothorax reevesii*	LC
				*Gymnothorax richardsonii*	LC
			*Strophidon*	*Strophidon dorsalis*	LC
				*Strophidon sathete*	LC
		Ophichthidae	*Scolecenchelys*	*Scolecenchelys macroptera*	LC
			*Neenchelys*	*Neenchelys parvipectoralis*	LC
			*Muraenichthys*	*Muraenichthys hattae*	DD
			*Ophichthus*	*Ophichthus apicalis*	LC
			*Pisodonophis*	*Pisodonophis boro*	LC
				*Pisodonophis cancrivorus*	LC
			*Brachysomophis*	*Brachysomophis crocodilinus*	LC
			*Caecula*	*Caecula pterygera*	DD
		Muraenesocidae	*Muraenesox*	*Muraenesox bagio*	LC
				*Muraenesox cinereus*	LC
			*Congresox*	*Congresox talabonoides*	LC
		Congridae	*Uroconger*	*Uroconger lepturus*	LC
			*Gnathophis*	*Gnathophis heterognathos*	LC
			*Rhynchoconger*	*Rhynchoconger ectenurus*	LC
			*Ariosoma*	*Ariosoma majus*	NE
		Moringuidae	*Moringua*	*Moringua macrocephalus*	DD
		Nettastomatidae	*Saurenchelys*	*Saurenchelys fierasfer*	LC
	Clupeiformes	Engraulidae	*Setipinna*	*Setipinna tenuifilis*	DD
			*Stolephorus*	*Stolephorus balinensis*	NE
				*Stolephorus chinensis*	LC
				*Stolephorus indicus*	LC
				*Stolephorus waitei*	DD
			*Thryssa*	*Thryssa adelae*	DD
				*Thryssa chefuensis*	DD
				*Thryssa dussumieri*	LC
				*Thryssa hamiltonii*	LC
				*Thryssa setirostris*	LC
			*Encrasicholina*	*Encrasicholina punctifer*	LC
		Pristigasteridae	*Ilisha*	*Ilisha elongata*	LC
				*Ilisha melastoma*	LC
		Clupeidae	*Sardinella*	*Sardinella fimbriata*	LC
				*Sardinella hualiensis*	LC
			*Nematalosa*	*Nematalosa nasus*	LC
			*Anodontostoma*	*Anodontostoma chacunda*	LC
	Siluriformes	Ariidae	*Arius*	*Arius arius*	LC
			*Plicofollis*	*Plicofollis nella*	NE
		Plotosidae	*Plotosus*	*Plotosus lineatus*	LC
	Aulopiformes	Synodontidae	*Synodus*	*Synodus fuscus*	LC
				*Synodus hoshinonis*	LC
				*Synodus jaculum*	LC
				*Synodus macrops*	LC
				*Synodus rubromarmoratus*	LC
				*Synodus tectus*	LC
				*Synodus variegatus*	LC
			*Trachinocephalus*	*Trachinocephalus myops*	LC
			*Harpadon*	*Harpadon nehereus*	NT
			*Saurida*	*Saurida elongata*	LC
				*Saurida filamentosa*	LC
				*Saurida gracilis*	LC
				*Saurida tumbil*	LC
				*Saurida undosquamis*	LC
	Myctophiformes	Myctophidae	*Benthosema*	*Benthosema fibulatum*	LC
				*Benthosema pterotum*	LC
	Gadiformes	Bregmacerotidae	*Bregmaceros*	*Bregmaceros lanceolatus*	NE
				*Bregmaceros mcclellandi*	NE
				*Bregmaceros pescadorus*	NE
				*Bregmaceros pseudolanceolatus*	NE
	Holocentriformes	Holocentridae	*Sargocentron*	*Sargocentron ittodai*	LC
				*Sargocentron rubrum*	LC
			*Ostichthys*	*Ostichthys sheni*	NE
	Ophidiiformes	Ophidiidae	*Brotula*	*Brotula multibarbata*	LC
			*Sirembo*	*Sirembo imberbis*	LC
	Scombriformes	Centrolophidae	*Psenopsis*	*Psenopsis anomala*	NE
		Stromateidae	*Pampus*	*Pampus chinensis*	NE
				*Pampus cinereus*	NE
				*Pampus echinogaster*	NE
				*Pampus minor*	NE
				*Pampus punctatissimus*	NE
		Ariommatidae	*Ariomma*	*Ariomma indica*	NE
		Scombridae	*Rastrelliger*	*Rastrelliger kanagurta*	LC
		Trichiuridae	*Lepturacanthus*	*Lepturacanthus savala*	NE
			*Trichiurus*	*Trichiurus brevis*	NE
				*Trichiurus japonicus*	LC
				*Trichiurus nanhaiensis*	NE
			*Tentoriceps*	*Tentoriceps cristatus*	NE
	Syngnathiformes	Dactylopteridae	*Dactyloptena*	*Dactyloptena gilberti*	LC
				*Dactyloptena orientalis*	LC
		Pegasidae	*Pegasus*	*Pegasus laternarius*	DD
		Mullidae	*Upeneus*	*Upeneus japonicus*	NE
				*Upeneus spottocaudalis*	NE
				*Upeneus sulphureus*	LC
				*Upeneus tragula*	LC
			*Parupeneus*	*Parupeneus heptacanthus*	LC
			*Mulloidichthys*	*Mulloidichthys vanicolensis*	LC
		Callionymidae	*Callionymus*	*Callionymus belcheri*	NE
				*Callionymus curvicornis*	LC
				*Callionymus hindsii*	NE
			*Repomucenus*	*Repomucenus beniteguri*	LC
				*Repomucenus huguenini*	NE
				*Repomucenus planus*	NE
				*Repomucenus virgis*	NE
			*Calliurichthys*	*Calliurichthys japonicus*	NE
			*Dactylopus*	*Dactylopus dactylopus*	LC
		Syngnathidae	*Halicampus*	*Halicampus grayi*	LC
				*Halicampus spinirostris*	LC
			*Hippocampus*	*Hippocampus mohnikei*	VU
				*Hippocampus trimaculatus*	VU
			*Trachyrhamphus*	*Trachyrhamphus serratus*	DD
		Fistulariidae	*Fistularia*	*Fistularia commersonii*	LC
				*Fistularia petimba*	LC
		Solenostomidae	*Solenostomus*	*Solenostomus armatus*	LC
	Kurtiformes	Apogonidae	*Ostorhinchus*	*Ostorhinchus doederleini*	LC
				*Ostorhinchus fasciatus*	LC
				*Ostorhinchus kiensis*	LC
				*Ostorhinchus semilineatus*	NE
			*Jaydia*	*Jaydia arafurae*	NE
				*Jaydia carinatus*	NE
				*Jaydia lineata*	LC
				*Jaydia novaeguineae*	LC
				*Jaydia* sp.	NE
				*Jaydia striata*	LC
				*Jaydia striatodes*	LC
				*Jaydia truncata*	LC
			*Verulux*	*Verulux cypselurus*	LC
		Trichonotidae	*Trichonotus*	*Trichonotus setiger*	LC
	Gobiiformes	Gobiidae	*Oxyurichthys*	*Oxyurichthys auchenolepis*	LC
				*Oxyurichthys ophthalmonema*	LC
			*Trypauchen*	*Trypauchen taenia*	NE
				*Trypauchen vagina*	LC
			*Paratrypauchen*	*Paratrypauchen microcephalus*	LC
			*Amblyotrypauchen*	*Amblyotrypauchen arctocephalus*	LC
			*Parachaeturichthys*	*Parachaeturichthys polynema*	LC
			*Valenciennea*	*Valenciennea wardii*	LC
			*Egglestonichthys*	*Egglestonichthys bombylios*	LC
				*Egglestonichthys melanoptera*	LC
			*Gobiodon*	*Gobiodon okinawae*	LC
			*Cryptocentrus*	*Cryptocentrus russus*	NE
			*Myersina*	*Myersina filifer*	LC
		Eleotridae	*Butis*	*Butis koilomatodon*	LC
	Carangiformes	Sphyraenidae	*Sphyraena*	*Sphyraena flavicauda*	LC
				*Sphyraena pinguis*	LC
				*Sphyraena putnamae*	LC
		Lactariidae	*Lactarius*	*Lactarius lactarius*	NE
		Polynemidae	*Polydactylus*	*Polydactylus sextarius*	NE
		Citharidae	*Brachypleura*	*Brachypleura novaezeelandiae*	LC
			*Citharoides*	*Citharoides macrolepidotus*	LC
		Bothidae	*Laeops*	*Laeops kitaharae*	LC
				*Laeops parviceps*	LC
			*Arnoglossus*	*Arnoglossus aspilos*	LC
				*Arnoglossus macrolophus*	LC
				*Arnoglossus polyspilus*	LC
				*Arnoglossus tapeinosoma*	DD
				*Arnoglossus tenuis*	LC
			*Engyprosopon*	*Engyprosopon grandisquama*	LC
				*Engyprosopon latifrons*	DD
				*Engyprosopon maldivense*	NE
				*Engyprosopon multisquama*	LC
			*Crossorhombus*	*Crossorhombus azureus*	LC
				*Crossorhombus kanekonis*	NE
			*Psettina*	*Psettina gigantea*	LC
				*Psettina iijimae*	LC
			*Parabothus*	*Parabothus taiwanensis*	LC
			*Grammatobothus*	*Grammatobothus krempfi*	DD
			*Japonolaeops*	*Japonolaeops dentatus*	NE
			*Bothus*	*Bothus myriaster*	LC
			*Asterorhombus*	*Asterorhombus intermedius*	LC
		Paralichthyidae	*Pseudorhombus*	*Pseudorhombus malayanus*	LC
				*Pseudorhombus oligodon*	LC
				*Pseudorhombus triocellatus*	LC
			*Tarphops*	*Tarphops oligolepis*	LC
		Samaridae	*Samariscus*	*Samariscus japonicus*	DD
				*Samariscus latus*	LC
			*Samaris*	*Samaris cristatus*	LC
		Soleidae	*Zebrias*	*Zebrias quagga*	LC
				*Zebrias zebrinus*	DD
			*Solea*	*Solea ovata*	LC
			*Aesopia*	*Aesopia cornuta*	LC
			*Aseraggodes*	*Aseraggodes kobensis*	LC
			*Pardachirus*	*Pardachirus pavoninus*	LC
		Cynoglossidae	*Symphurus*	*Symphurus* sp.	NE
			*Cynoglossus*	*Cynoglossus arel*	DD
				*Cynoglossus kopsii*	LC
				*Cynoglossus nanhaiensis*	DD
				*Cynoglossus nigropinnatus*	LC
				*Cynoglossus ochiaii*	LC
				*Cynoglossus oligolepis*	DD
				*Cynoglossus puncticeps*	LC
				*Cynoglossus quadrilineatus*	LC
				*Cynoglossus roulei*	NE
				*Cynoglossus semilaevis*	DD
		Carangidae	*Alepes*	*Alepes kleinii*	LC
				*Alepes melanoptera*	LC
			*Decapterus*	*Decapterus maruadsi*	LC
				*Decapterus tabl*	LC
			*Megalaspis*	*Megalaspis cordyla*	LC
			*Carangoides*	*Carangoides armatus*	LC
				*Carangoides chrysophrys*	LC
				*Carangoides malabaricus*	LC
			*Selar*	*Selar crumenophthalmus*	LC
			*Alectis*	*Alectis indica*	LC
			*Parastromateus*	*Parastromateus niger*	LC
			*Atropus*	*Atropus atropos*	LC
			*Kaiwarinus*	*Kaiwarinus equula*	NE
			*Trachurus*	*Trachurus japonicus*	NT
			*Caranx*	*Caranx ignobilis*	LC
				*Caranx sexfasciatus*	LC
	Cichliformes	Opistognathidae	*Opistognathus*	*Opistognathus evermanni*	LC
		Pomacentridae	*Chromis*	*Chromis fumeus*	LC
			*Teixeirichthys*	*Teixeirichthys jordani*	LC
	Mugiliformes	Mugilidae	*Moolgarda*	*Moolgarda cunnesius*	NE
				*Moolgarda pedaraki*	NE
				*Moolgarda perusii*	LC
			*Planiliza*	*Planiliza macrolepis*	LC
	Blenniiformes	Blenniidae	*Xiphasia*	*Xiphasia setifer*	LC
			*Plagiotremus*	*Plagiotremus spilistius*	LC
	Perciformes	Serranidae	*Diploprion*	*Diploprion bifasciatum*	LC
			*Tosana*	*Tosana niwae*	NE
			*Epinephelus*	*Epinephelus areolatus*	LC
				*Epinephelus awoara*	DD
				*Epinephelus bleekeri*	DD
				*Epinephelus coioides*	LC
				*Epinephelus craigi*	NE
				*Epinephelus latifasciatus*	LC
				*Epinephelus quoyanus*	LC
				*Epinephelus sexfasciatus*	LC
			*Cephalopholis*	*Cephalopholis boenak*	LC
			*Triso*	*Triso dermopterus*	NE
			*Chelidoperca*	*Chelidoperca hirundinacea*	NE
				*Chelidoperca margaritifera*	NE
		Labridae	*Suezichthys*	*Suezichthys gracilis*	LC
			*Leptojulis*	*Leptojulis lambdastigma*	DD
			*Xiphocheilus*	*Xiphocheilus typus*	LC
		Uranoscopidae	*Uranoscopus*	*Uranoscopus japonicus*	LC
				*Uranoscopus oligolepis*	LC
				*Uranoscopus tosae*	NE
		Pinguipedidae	*Parapercis*	*Parapercis cylindrica*	LC
				*Parapercis lutevittata*	NE
				*Parapercis ommatura*	NE
				*Parapercis pulchella*	LC
				*Parapercis sexfasciata*	NE
				*Parapercis snyderi*	LC
		Ammodytidae	*Bleekeria*	*Bleekeria viridianguilla*	NE
		Platycephalidae	*Inegocia*	*Inegocia japonica*	LC
			*Grammoplites*	*Grammoplites knappi*	NE
			*Sunagocia*	*Sunagocia arenicola*	LC
			*Suggrundus*	*Suggrundus meerdervoortii*	NE
			*Platycephalus*	*Platycephalus indicus*	DD
			*Cociella*	*Cociella punctata*	LC
			*Elates*	*Elates ransonnettii*	NE
			*Onigocia*	*Onigocia spinosa*	LC
			*Thysanophrys*	*Thysanophrys chiltonae*	LC
			*Rogadius*	*Rogadius asper*	LC
			*Kumococius*	*Kumococius rodericensis*	LC
		Synanceiidae	*Apistus*	*Apistus carinatus*	NE
			*Erisphex*	*Erisphex pottii*	LC
				*Erisphex simplex*	NE
			*Paracentropogon*	*Paracentropogon rubripinnis*	LC
			*Ablabys*	*Ablabys macracanthus*	LC
			*Richardsonichthys*	*Richardsonichthys leucogaster*	NE
			*Choridactylus*	*Choridactylus multibarbus*	LC
			*Inimicus*	*Inimicus cuvieri*	NE
			*Trachicephalus*	*Trachicephalus uranoscopus*	LC
			*Minous*	*Minous monodactylus*	LC
				*Minous pictus*	NE
				*Minous pusillus*	NE
		Triglidae	*Lepidotrigla*	*Lepidotrigla abyssalis*	NE
				*Lepidotrigla alata*	NE
				*Lepidotrigla japonica*	NE
			*Chelidonichthys*	*Chelidonichthys spinosus*	LC
		Scorpaenidae	*Neomerinthe*	*Neomerinthe megalepis*	LC
			*Scorpaenopsis*	*Scorpaenopsis neglecta*	LC
				*Scorpaenopsis ramaraoi*	LC
			*Pterois*	*Pterois lunulata*	LC
				*Pterois russelii*	LC
			*Parapterois*	*Parapterois heterurus*	LC
			*Dendrochirus*	*Dendrochirus brachypterus*	LC
			*Brachypterois*	*Brachypterois serrulata*	LC
			*Setarches*	*Setarches longimanus*	NE
			*Sebastiscus*	*Sebastiscus marmoratus*	NE
	Centrarchiformes	Terapontidae	*Terapon*	*Terapon jarbua*	LC
				*Terapon theraps*	LC
			*Pelates*	*Pelates quadrilineatus*	NE
		Cirrhitidae	*Cirrhitichthys*	*Cirrhitichthys aureus*	LC
	Acropomatiformes	Champsodontidae	*Champsodon*	*Champsodon longipinnis*	NE
				*Champsodon snyderi*	NE
		Acropomatidae	*Acropoma*	*Acropoma japonicum*	NE
	Acanthuriformes	Chaetodontidae	*Roa*	*Roa modesta*	NE
			*Heniochus*	*Heniochus diphreutes*	LC
		Leiognathidae	*Leiognathus*	*Leiognathus equula*	NE
			*Photopectoralis*	*Photopectoralis bindus*	LC
			*Eubleekeria*	*Eubleekeria jonesi*	LC
			*Deveximentum*	*Deveximentum ruconius*	NE
			*Photolateralis*	*Photolateralis stercorarius*	LC
			*Equulites*	*Equulites berbis*	LC
			*Gazza*	*Gazza minuta*	LC
				*Gazza rhombea*	LC
			*Nuchequula*	*Nuchequula mannusella*	LC
				*Nuchequula nuchalis*	NE
			*Karalla*	*Karalla daura*	LC
		Sciaenidae	*Johnius*	*Johnius amblycephalus*	LC
				*Johnius belangerii*	LC
				*Johnius borneensis*	LC
				*Johnius carouna*	LC
				*Johnius fasciatus*	LC
				*Johnius grypotus*	LC
				*Johnius taiwanensis*	LC
				*Johnius trewavasae*	LC
			*Pennahia*	*Pennahia anea*	LC
				*Pennahia argentata*	LC
				*Pennahia macrocephalus*	LC
				*Pennahia pawak*	LC
			*Larimichthys*	*Larimichthys crocea*	CR
			*Nibea*	*Nibea semifasciata*	DD
			*Dendrophysa*	*Dendrophysa russelii*	LC
			*Chrysochir*	*Chrysochir aureus*	LC
			*Otolithes*	*Otolithes ruber*	LC
		Sparidae	*Evynnis*	*Evynnis cardinalis*	EN
			*Acanthopagrus*	*Acanthopagrus latus*	DD
				*Acanthopagrus pacificus*	LC
				*Acanthopagrus schlegelii*	LC
				*Acanthopagrus taiwanensis*	DD
		Nemipteridae	*Nemipterus*	*Nemipterus bathybius*	LC
				*Nemipterus furcosus*	LC
				*Nemipterus japonicus*	LC
				*Nemipterus marginatus*	LC
				*Nemipterus nemurus*	LC
				*Nemipterus peronii*	LC
				*Nemipterus virgatus*	VU
				*Nemipterus zysron*	LC
			*Scolopsis*	*Scolopsis vosmeri*	LC
			*Pentapodus*	*Pentapodus setosus*	LC
		Siganidae	*Siganus*	*Siganus canaliculatus*	LC
				*Siganus fuscescens*	LC
		Sillaginidae	*Sillago*	*Sillago aeolus*	NE
				*Sillago asiatica*	NE
				*Sillago ingenuua*	NE
				*Sillago japonica*	LC
				*Sillago sihama*	LC
		Ephippidae	*Ephippus*	*Ephippus orbis*	NE
		Haemulidae	*Pomadasys*	*Pomadasys grunniens*	NE
				*Pomadasys maculatus*	LC
			*Parapristipoma*	*Parapristipoma trilineatum*	LC
		Lutjanidae	*Pristipomoides*	*Pristipomoides filamentosus*	LC
				*Pristipomoides multidens*	LC
			*Lutjanus*	*Lutjanus johnii*	LC
				*Lutjanus lutjanus*	LC
				*Lutjanus malabaricus*	LC
				*Lutjanus monostigma*	LC
				*Lutjanus russellii*	LC
			*Pterocaesio*	*Pterocaesio chrysozona*	LC
		Cepolidae	*Acanthocepola*	*Acanthocepola krusensternii*	NE
				*Acanthocepola limbata*	NE
		Malacanthidae	*Branchiostegus*	*Branchiostegus albus*	NT
				*Branchiostegus argentatus*	NT
		Priacanthidae	*Priacanthus*	*Priacanthus macracanthus*	LC
		Lobotidae	*Hapalogenys*	*Hapalogenys analis*	NE
		Gerreidae	*Gerres*	*Gerres filamentosus*	LC
				*Gerres oblongus*	LC
				*Gerres oyena*	LC
				*Gerres septemfasciatus*	NE
		Lethrinidae	*Lethrinus*	*Lethrinus lentjan*	LC
		Scatophagidae	*Scatophagus*	*Scatophagus argus*	LC
		Drepaneidae	*Drepane*	*Drepane punctata*	LC
	Lophiiformes	Lophiidae	*Lophiomus*	*Lophiomus setigerus*	LC
		Ogcocephalidae	*Halieutaea*	*Halieutaea indica*	LC
		Antennariidae	*Antennarius*	*Antennarius hispidus*	LC
				*Antennarius nummifer*	LC
				*Antennarius striatus*	LC
	Tetraodontiformes	Tetraodontidae	*Lagocephalus*	*Lagocephalus gloveri*	DD
				*Lagocephalus inermis*	LC
				*Lagocephalus spadiceus*	LC
				*Lagocephalus suezensis*	LC
			*Takifugu*	*Takifugu oblongus*	LC
				*Takifugu poecilonotus*	LC
			*Torquigener*	*Torquigener brevipinnis*	LC
			*Canthigaster*	*Canthigaster rivulata*	LC
		Diodontidae	*Diodon*	*Diodon holocanthus*	LC
		Monacanthidae	*Paramonacanthus*	*Paramonacanthus pusillus*	LC
			*Monacanthus*	*Monacanthus chinensis*	LC
			*Thamnaconus*	*Thamnaconus hypargyreus*	LC
			*Aluterus*	*Aluterus monoceros*	LC
			*Anacanthus*	*Anacanthus barbatus*	LC
			*Stephanolepis*	*Stephanolepis cirrhifer*	LC

Notes: CR, Critically Endangered; EN, Endangered; VU, Vulnerable; NT, Near Threatened; LC, Least Concern; DD, Data Deficient; NE, Not Evaluated.
